# Dynamic Characteristic Model of Giant Magnetostrictive Transducer with Double Terfenol-D Rods

**DOI:** 10.3390/mi14061103

**Published:** 2023-05-24

**Authors:** Yafang Li, Xia Dong, Xiaodong Yu

**Affiliations:** School of Information and Automation Engineering, Qilu University of Technology (Shandong Academy of Sciences), Jinan 250353, China; dongxia6078@163.com (X.D.); xiaodongyu2001@qlu.edu.cn (X.Y.)

**Keywords:** giant magnetostrictive transducer, high frequency, output displacement, acceleration

## Abstract

Giant magnetostrictive transducer can be widely used in active vibration control, micro-positioning mechanism, energy harvesting system, and ultrasonic machining. Hysteresis and coupling effects are present in transducer behavior. The accurate prediction of output characteristics is critical for a transducer. A dynamic characteristic model of a transducer is proposed, by providing a modeling methodology capable of characterizing the nonlinearities. To attain this objective, the output displacement, acceleration, and force are discussed, the effects of operating conditions on the performance of Terfenol-D are studied, and a magneto-mechanical model for the behavior of transducer is proposed. A prototype of the transducer is fabricated and tested to verify the proposed model. The output displacement, acceleration, and force have been theoretically and experimentally studied at different working conditions. The results show that, the displacement amplitude, acceleration amplitude, and force amplitude are about 49 μm, 1943 m/s^2^, and 20 N. The error between the model and experimental results are 3 μm, 57 m/s^2^, and 0.2 N. Calculation results and experimental results show a good agreement.

## 1. Introduction

Magnetostriction is a physical phenomenon of ferromagnetic materials in which the state of mechanical strain depends upon the direction and strength of its magnetization. Terfenol-D (Tb_0.27_Dy_0.73_Fe_2_) is a kind of magnetostrictive material. Terfenol-D can accept high power electrical input and convert it to high frequency power. Terfenol-D exhibits magnetostricion coefficients along principal crystallographic directions of $\lambda_{111}=1600\times 10^{-6}$. Terfenol-D shows the largest room temperature magnetostriction of any known magnetostrictive material. In addition, Terfenol-D has the advantages of high energy density, fast response speed, and control accuracy for the application in intelligent devices. Owing to its significant characteristics, it can be widely used in active vibration control, micro-positioning mechanism, energy harvesting system, and ultrasonic machining [1,2,3,4]. When used as a giant magnetostrictive transducer driver element, Terfenol-D is capable of generating large output characteristics under the application of exciting magnetic fields, a bias magnetic field, and a prestress. Owing to the coupling effect between the electric, magnetic, thermal, and structural fields, modeling the nonlinear behavior of giant magnetostrictive transducer is further complicated. Based on magnetostrictive material models and magnetostrictive device models, numerous modeling techniques have been developed. However, comprehensive models capable of providing accurate simulation of magnetostrictive performance are still lacking.

For the research on the modeling nonlinear constitutive relationships for magnetostrictive materials, Xiao et al. proposed a 1D nonlinear elasto-thermo-magnetic constitutive model, which can reflect the influence of varied stress and temperature on the effective magnetic field on the static and dynamic magnetostrictive effect [5]. On the basis of Jiles-Atherton hysteresis theory, Zheng et al., established a dynamic hysteresis constitutive relation considering the energy loss arising from a time-varying field [6]. Talebian et al. studied classical and excess eddy currents losses of Terfenol-D, and presented a model using the combination of Preisach and Hyperbolic Tangent models, to predict the magnetic hysteresis of Terfenol-D at different frequencies [7,8]. However, the models have not established the relationships between the material constitutive model and the output characteristics of devices.

Currently, a variety of output characteristics model for magnetostrictive devices have been studied with two types. One is linear magneto-mechanical models [9,10,11,12]. The other is finite element models [13,14,15,16]. Braghin et al. developed a linear model of a transducer, which can predict the dynamic behavior in a range of frequencies between 40 Hz and 2 kHz [9]. On the basis of the linear magneto-mechanical theory, Zhang et al., proposed a nonlinear constitutive model of an actuator [10]. According to linear system theory, Xue et al., presented a single degree of freedom second-order system, but the model does not take eddy current losses of the material at high frequencies into consideration. The model is suitable for the condition where the input signal frequencies are less than 200 Hz [11]. Scheidler et al. presented a linear model of the fully coupled electromechanical behavior of a transducer. The magnetic characteristic parameters of the material have been studied at the frequency range of 100–800 Hz [12]. Due to the nonlinear characteristics of the Terfenol-D at high frequencies, the linear models are not applicable. By contrast, the most typical finite element model is proposed to describe the magnetostriction mechanisms of the devices. Moreover, because of including the use of few physically related material parameters and simplicity of use, this model is very practical in engineering applications.

Huang et al., presented a numerical dynamic and magneto-mechanical strong coupled model of magnetostrictive devices [13], but nonlinear material properties have not been described. Chakrabarti et al. developed a lumped parameter model considering nonlinear Terfenol-D material response to model the dynamic mechanical response. The output response was calculated at a frequency of 1 kHz [14]. Li et al. developed a comprehensive model that can describe the current and displacement responses of magnetostrictive transducers. The output response was calculated at a frequency of 230 Hz [15]. Wang et al. designed a magnetostrictive transducer with a resonant frequency of 2 kHz and analyzed the resonant frequency using finite element method [16]. Authors of [13,14,15,16] have established numerical models for magnetostrictive devices at low-frequency excitation. Ma et al. designed a separated rotary giant magnetostrictive system. It was found to work with an ultrasonic amplitude of 10 μm [17]. Fang et al. designed an ultrasonic transducer with 15.2 kHz working frequency and 8 μm ultrasonic amplitude [18]. Zhou et al. presented a mathematical model for optimum impedance compensation of an ultrasonic transducer [19]. However, the references [17,18,19] cannot predict the frequency-dependent output characteristics (displacement, acceleration, and force) and coupling nonlinear constitutive behavior of the Terfenol-D at various operating conditions. Therefore, it is necessary to establish a more suitable and concise dynamic characteristic model for giant magnetostrictive transducers in engineering applications.

In this article, a giant magnetostrictive transducer which has a large output displacement and force has been fabricated. A dynamic characteristic model of the transducer is proposed, by providing a modeling methodology capable of characterizing the nonlinearities. The output displacement, acceleration, and force are measured and analyzed at different exciting magnetic field and frequencies. The results can predict the frequency-dependent output characteristics of a Terfenol-D device at high frequency.

## 2. Dynamic Characteristic Model of Giant Magnetostrictive Transducer Considering Magneto-Mechanical Coupling Effect

### 2.1. Finite Element Model for Giant Magnetostrictive Transducer

The modeling of electromagnetic field is described from the Maxwell equations [14].

(1) $$\begin{array}{l} \nabla \times H=J+\partial D/\partial t\\ \nabla \times E=-\partial B/\partial t\\ \nabla \cdot D=\rho_{e}\\ \nabla \cdot B=0 \end{array}$$

where ***H*** is the magnetic field, ***E*** is the electric field, ***D*** is the electric displacement, ***B*** is the magnetic flux density, ***J*** is the conduction current density, ∂D/∂t is the displacement current density, and *ρ_e_* is the free charge volume density. In order to make sure the Maxwell equations have definite solutions, the electromagnetic constitutive relations must be introduced.

(2) $$\begin{array}{l} B=\mu H\\ J=\kappa E\\ D=\varepsilon_{0}E \end{array}$$

where *μ* is the permeability, κ is the conductivity, and $\varepsilon_{0}$ is the dielectric constant. The magnetic vector potential ***A*** and the electrical scalar potential *U* are introduced to simplify calculation, as shown in Equation (3).

(3) $$\begin{array}{l} B=\nabla \times A\\ E=-\nabla U-\frac{\partial A}{\partial t} \end{array}$$

Because the effect of ∂B/∂t is far greater than that of ∂D/∂t, ∂D/∂t can be neglected. According to the Maxwell equations, the governing equation of the electromagnetic field can be mathematically modeled as follows:

(4) $$\kappa \cdot \frac{\partial A}{\partial t}+\nabla \times H=J$$

According to the Galerkin weighted residual method, the weighted quantity δA is multiplied and integrated in the whole domain. The weak form of the electromagnetic field equation is

(5) $$\int_{V}\kappa \cdot \frac{\partial A}{\partial t}\cdot \delta AdV+\int_{V}H\cdot \left(\nabla \times \delta A\right)dV=\int_{V}J\cdot \delta AdV+\int_{\partial V}\left(H\times n\right)\cdot \delta Ad\partial V$$

where *V* is the volume, *∂V* is the boundary enclosing the volume, and ***n*** is the normal vector of the boundary surface. Based on the Newton’s second law, the mechanical field can be mathematically modeled as follows:

(6) $$\nabla \cdot t^{\hat{n}}+b=\rho \ddot{u}$$

where $t^{\hat{n}}$ is the stress tensor, *ρ* is the density, ***b*** is the volume force, and ***u*** is the displacement vector. According to the Galerkin weighted residual method, the weighted quantity δu is multiplied and integrated in the whole domain. The weak form of the mechanical field equation is

(7) $$\int_{V}t^{\hat{n}}\delta udV+\int_{V}\rho \ddot{u}\delta udV=\int_{\partial V}t^{\hat{n}}\delta ud\partial V+\int_{V}b\delta udV$$

According to the Equations (4)–(7), the governing equations of the electromagnetic field and the mechanical field for a giant magnetostrictive transducer are established. Based on the governing equations, a 3-D simulation of the whole transducer working process is quantified using finite element software at different initial conditions, boundary conditions, and driving currents.

**Figure 1:**
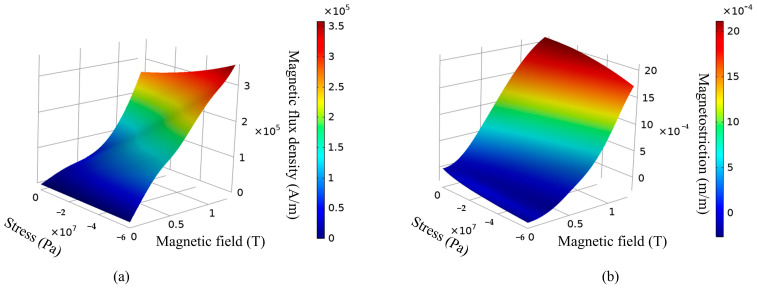
(**a**) The ***B****-**H*** curve of the Terfenol-D, (**b**) the *λ-**H*** curve of the Terfenol-D.

The nonlinear magnetic hysteresis behavior of the Terfenol-D rod is modeled by using a ***B****-**H*** curve to specify the magnetic constitutive relation. The nonlinear magneto-mechanical coupling effects of the magnetostrictive behavior is modeled by using a *λ-**H*** curve. The Terfenol-D constitutive law is coded up as Matlab functions [20]. The ***B****-**H*** curve and *λ-**H*** curve of the Terfenol-D are shown in Figure 1. The main parameters are given in Table 1.

### 2.2. The Structure and Materials of Giant Magnetostrictive Transducer

**Figure 2:**
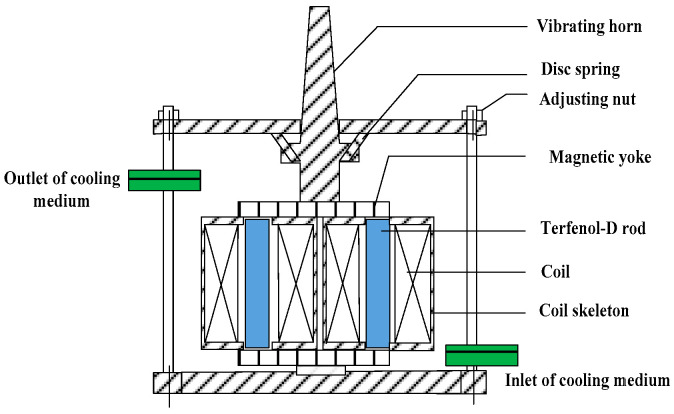
The structure of the giant magnetostrictive transducer.

A giant magnetostrictive transducer is designed and analyzed. The main part of the giant magnetostrictive transducer is shown in Figure 2. The giant magnetostrictive transducer consists of two Terfenol-D rods (Tb_0.27_Dy_0.73_Fe_2_), driving coils, adjusting nut, vibrating horn, magnetic yoke, and shell. The magnetostrictive material is placed in the core that works as an actuator when a magnetic field is applied by passing a current through the driving coil. The Terfenol-D rod is surrounded by driving coils. The magnetic circuit is closed through magnetic yoke. The structure parameters of the giant magnetostrictive transducer are presented in Table 2 [21].

In the design of magnetic circuit, the relative permeability and structure of the magnetic yoke affect the magnetic flux density of the Terfenol-D rod. In this paper, silicon steel sheet with high relative permeability (*u_r_* = 7000–10,000) is selected as a material of the magnetic yoke. The magnetic yoke width is 16 mm. The magnetic yoke length is 60 mm.

### 2.3. Finite Element Calculation of the Dynamic Characteristic Model

**Figure 3:**
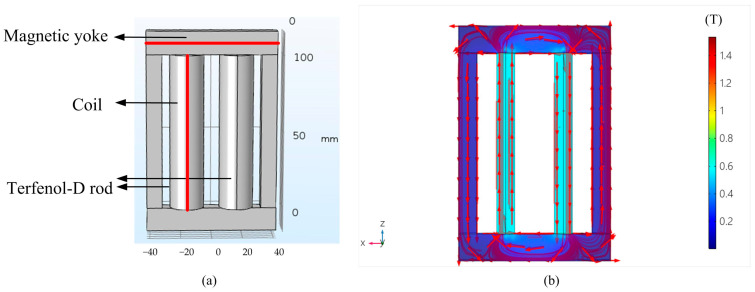
The simulation of the transducer (**a**) the structure, (**b**) the distribution of magnetic flux density.

By the Equations (4)–(7), the magnetic field and dynamic output characteristics of the giant magnetostrictive transducer are calculated and analyzed. In order to obtain concise representation of results, the shell, the vibrating horn, and the coils of the transducer are hidden in post-processing, as shown in Figure 3a. Magnetic circuit design needs to improve magnetic flux density and the uniformity of magnetic flux density of the Terfenol-D rod, and reduce magnetic losses of the magnetic circuit. The reason is that the magnetic leakage will cause serious distortion in a driving current.

The magnetic flux density distribution of the transducer is calculated in the conditions of 10 MPa prestress and 8 A current, as shown in Figure 3b. The red arrows represent the direction of the magnetic flux density. The magnetic circuit structure of the transducer is designed to create a closed magnetic flux path thereby minimizing flux leakage. It is mainly composed of the magnetic yoke and the Terfenol-D rod. Figure 3b shows the magnetic flux concentration in the magnetostrictive core due to the closed magnetic path. The magnetic flux density in the rods is mostly uniform. Fringe effects can be seen at both ends of the rod where majority of the magnetic flux density is forced to curl into the magnetic circuit structure.

**Figure 4:**
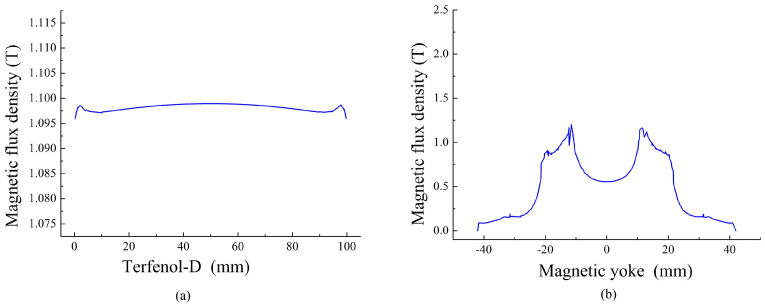
(**a**) The magnetic flux density of the Terfenol-D rod, (**b**) the magnetic flux density of the magnetic yoke.

The magnetic flux density in the internal central line of the Terfenol-D rod is shown in Figure 4a. The average magnetic flux density of the Terfenol-D rod is 1.097 T. In addition, the results show that the non-uniformity increases at both ends of the rod. Figure 4b shows the magnetic flux density in the magnetic yoke. The maximum magnetic flux density of the magnetic yoke is 1.2 T. The Terfenol-D rod’s position is in 20 mm and −20 mm. Through the analysis of the uniformity and the leakage of the magnetic flux density, it can be concluded that compared with the traditional single Terfenol-D rod transducer [11], the magnetic circuit structure of two Terfenol-D rods is more suitable for high frequency excitation.

**Figure 5:**
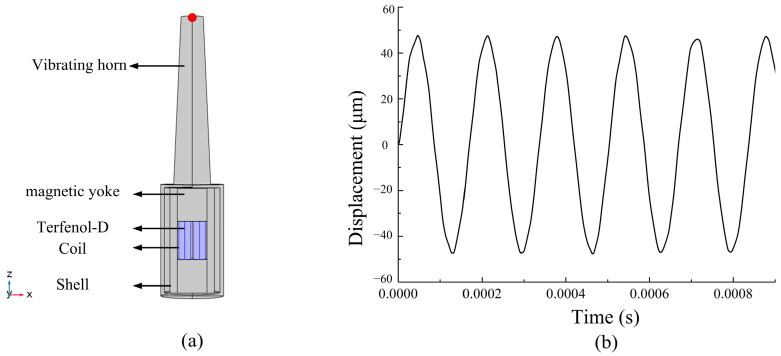
(**a**) The structure of the transducer, (**b**) the output displacement of the transducer.

**Figure 6:**
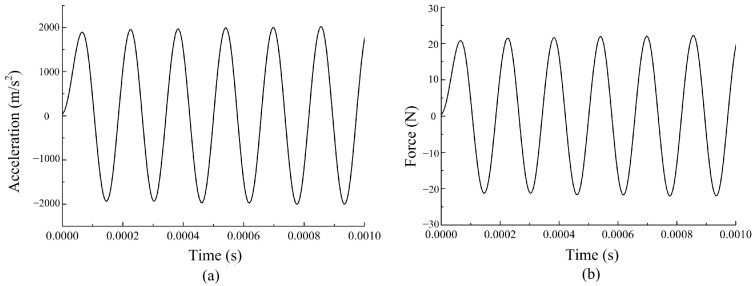
(**a**) The output acceleration of the transducer, (**b**) the output force of the transducer.

When prestress of the giant magnetostrictive transducer is set to 10 MPa and sinusoidal current of 2 A, 6.4 kHz is input into the transducer, and the simulation of displacement, acceleration, and force by the dynamic characteristic model is calculated, as shown in Figure 5 and Figure 6. The base point for the output displacement is set to 0 μm. We can see that the output displacement, acceleration, and force of the transducer are sinusoidal waves without distortion. The amplitude of the vibration displacement is 46 μm. The amplitude of the acceleration is 2000 m/s^2^. The amplitude of the force is 20.2 N.

The magnetic flux density, the output displacement, the output acceleration, and the output force are calculated and simulated, analyzing and summarizing the data, which can serve as a reference for future measurement and design of the transducer.

## 3. The Prototype of Giant Magnetostrictive Transducer and Experimental System

**Figure 7:**
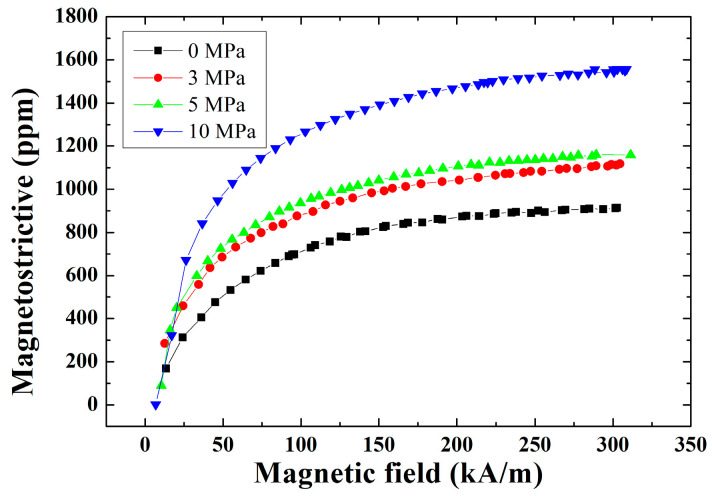
Magnetostrictive of Terfenol-D with different prestress.

**Figure 8:**
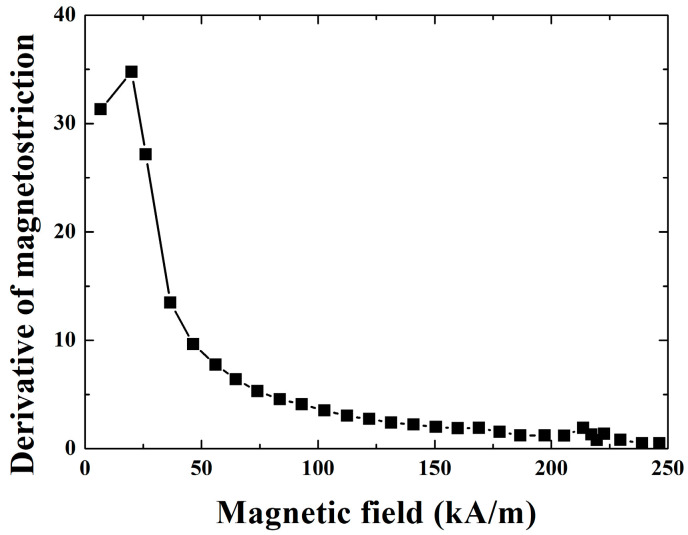
Derivative of magnetostriction (10 MPa prestress).

In order to improve the amplitude of the transducer, the prestress should be determined with the aim of maximizing the magnetostrictive property. The Terfenol-D rods are installed between the extension block of vibrating horn and the support plate through adjusting nut and disc spring. Adjusting disc spring and adjusting nut, the Terfenol-D can be worked under a designed prestress. The magnetostrictive curves of the Terfenol-D under different prestress are studied experimentally, as shown in Figure 7. It can be seen that when the prestress of the Terfenol-D is 10 MPa, the magnetostrictive property of the Terfenol-D is better. By calculating the slope of the magnetostrictive curve under 10 MPa prestress, the bias magnetic field can be determined, as shown in Figure 8. The maximum slope of the magnetostrictive curve is 34.77 when the magnetic field is 20 kA/m. In addition, the linearity of the magnetostrictive curve is better in the range of magnetic field *H* < 40 kA/m. The magnetic field corresponding to the midpoint of the linear section of the curve under certain prestress is determined as the bias magnetic field. According to these characteristics, the bias magnetic field *H*_0_ = 20 kA/m (*I*_0_ = 8 A) is properly selected.

**Figure 9:**
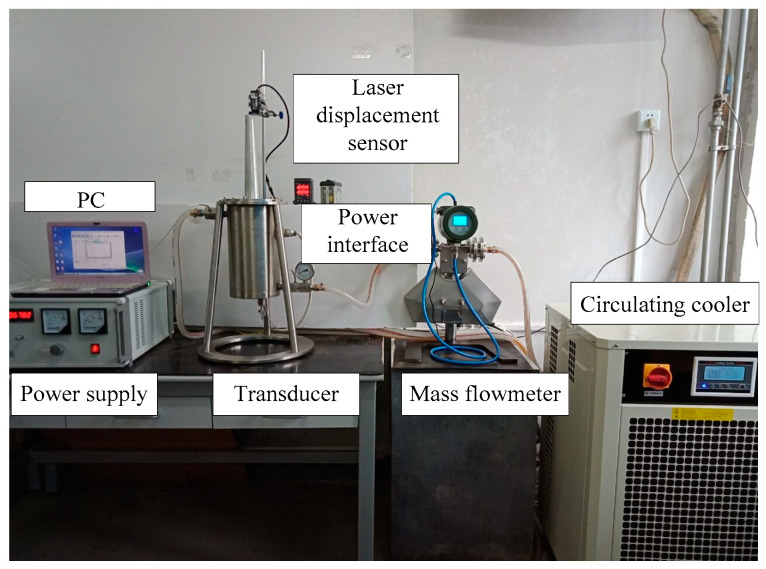
Experimental system of the giant magnetostrictive transducer.

The prototype of a giant magnetostrictive transducer and an experimental system of dynamic characteristics are shown in Figure 9. The experimental system of dynamic characteristics can measure the output displacement, acceleration, and force of the transducer. It is composed of the following components: giant magnetostrictive transducer, PC, power supply, mass flowmeter (YK-LK-10-FT51S, Yoke Instrument, Dalian, China), circulating cooler (Shanghai TOYO, Shanghai, China), displacement sensor (KEYENCE, Beijing, China), SQLab II vibration, and noise test system and force sensor (L1015, Yangzhou, China). The Terfenol-D transducer is a current-driven device. The power supply can provide AC excitation and DC bias. The AC is always applied to exert driven magnetic field, and the DC is employed to bias the Terfenol-D magnetically, which enables the transducer to operate in the linear region. The bias current is 8 A (*H*_0_ = 20 kA/m), and the amplitudes of the exciting current are 1 A and 2 A (*H*_ac_ = 2.5 kA/m and 5 kA/m).

The prediction accuracy of the magnetic energy losses is critical for the reliable generation of output characteristics. The hysteresis loss and eddy current loss have been studied [21]. Moreover, a cooling system must match the overall heat generated during operation. Based on the convection-heat-transfer theory and the thermal-compensation method, a temperature-control system is designed. The accuracy and feasibility of the proposed approach are tested by the calculating the results and the experimental ones. The cooling system is composed of circulating cooler, mass flowmeter, and active Terfenol-D rod cooling module. The forced cooling method is used for the cooling system of the transducer. The cooling medium is silicon oil. There are temperature sensors at the inlet and outlet of the transducer. The temperature sensors can record a real-time temperature in the active Terfenol-D rod cooling module. The flow velocity of silicone oil can be controlled by adjusting the circulating cooler. The mass flowmeter is used for monitoring the velocity of the silicone oil.

## 4. Results

**Figure 10:**
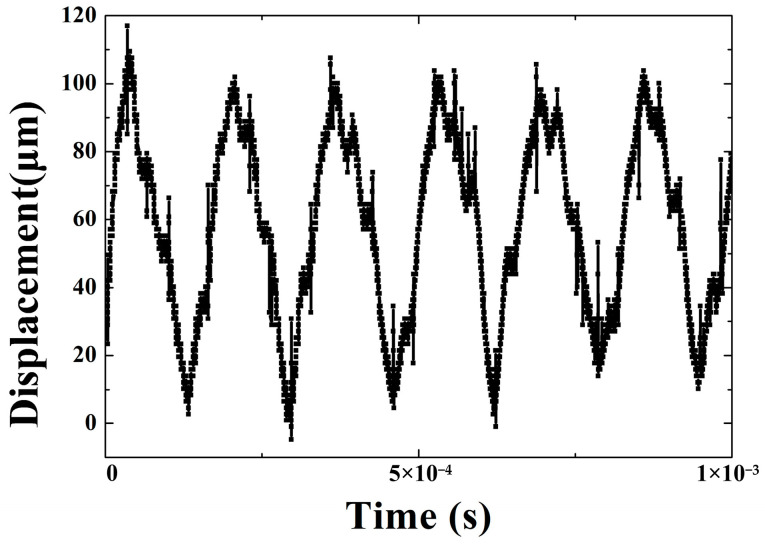
The displacement at 6.4 kHz.

The output displacement, acceleration, and force are studied by the experimental system. In addition, the relationship between output characteristics and driving frequency of the transducer is tested and analyzed. A real-time displacement at 2 A driving current and 6.4 kHz frequency is shown in Figure 10. The output displacement is measured by a displacement sensor. The experimental data directly displayed on a Tektronix DPO3014 oscilloscope are voltage values, and the displacement data are obtained by the corresponding relationship between the voltage and the displacement. The corresponding relationship is 1 V=20 μm. The results show that the average amplitude of output displacement is 49 μm. The value and shape of the curve in the Figure 10 is almost same as the one in the Figure 5b. Discrepancy between calculation displacement and experiment value is 3 μm.

**Figure 11:**
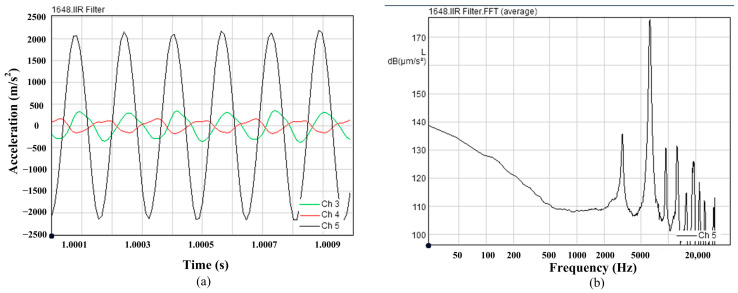
The acceleration of the transducer (**a**) real time acceleration, (**b**) frequency spectrum 0–20 kHz by FFT.

The output acceleration is measured by an acceleration sensor. The acceleration sensor can simultaneously measure a three-dimensional acceleration. The time history of the output acceleration is measured at 2 A driving current and 6.4 kHz frequency, as shown in Figure 11a. The three-dimensional directions are shown in the Figure 5a. The channel 3, channel 4, and channel 5 represent the x direction, y direction, and z direction in the Figure 11a. The acceleration in the z direction is much larger than that in the other directions, therefore the output acceleration in z direction is mainly analyzed. The value and the shape of the curve in the Figure 11a is almost same as the one in the Figure 6a. The amplitude of the acceleration is 1943 m/s^2^. Discrepancy between calculation value and experiment value is 2.8%. Results showed that the modeled responses compare well with experiments. These verify the correctness of the nonlinear dynamic model. Figure 11b shows the FFT results of the vibration acceleration at *f* = 6.4 kHz. The results show that the highest energy of the transducer is at 6.4 kHz frequency.

**Figure 12:**
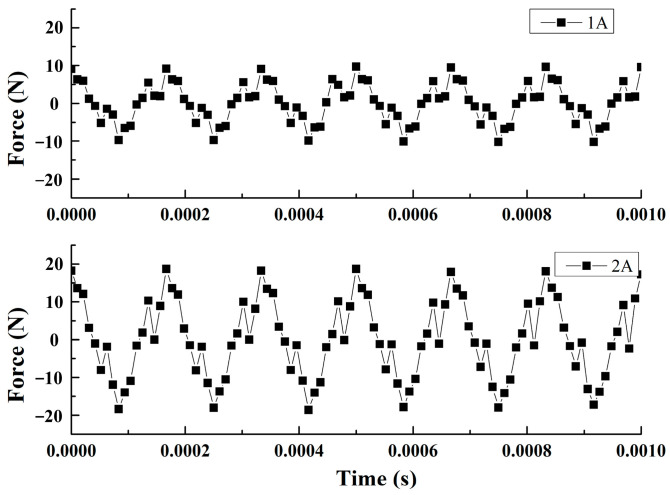
The output force of the transducer at 6.4 kHz frequency.

When the transducer is unloaded, the frequency is 6.4 kHz, and the driving current is 1 A and 2 A, the output forces of the transducer are shown in Figure 12. The curve of output force also varies in a sinusoidal wave. When the driving current are 1 A and 2 A, the output force amplitude of the transducer is 10 N and 20 N. When the driving current is 2 A, the amplitude of the output force calculated by the model is 20.2 N (Figure 6b), and the error between the calculated value and the experimental ones is 1%.

The output displacements at different frequencies are analyzed. The experiment conditions are as follows: the driving current is 1 A and 2 A, the bias current is 8 A, the cooling temperature of inlet is 20 °C, and the flow velocity of the silicone oil is 0.5 m/s. The frequencies are 5.6 kHz, 5.8 kHz, 6 kHz, 6.2 kHz, 6.4 kHz, 6.6 kHz, 6.8 kHz, and 7 kHz, respectively. The experimental results are exhibited in Table 3. With the frequency and driving current increasing, the displacement amplitude increases first and then decreases. The most displacement amplitude are 33 μm (*I* = 1 A) and 49 μm (*I* = 2 A) at 6.4 kHz driving frequency. The results show that the transducer has the best energy conversion rate when it operates at 6.4 kHz frequency.

In addition, when the frequency is 5.6 kHz and the driving current is 1 A, the temperature difference between the inlet and outlet is 1.1 °C. When the frequency is 7 kHz and the driving current is 2 A, the temperature difference rises to 3 °C. From this, it can be seen that the temperature difference between the inlet and outlet in the cooling circuit increases as the frequency and driving current increase. The reason is that the magnetic energy losses of the transducer will increase when the frequency increases. By controlling the flow rate of the silicone oil, the temperature difference can be controlled within a reasonable range, enabling the equipment on the best situation.

The output acceleration and force are analyzed at different frequencies. The results are shown in Table 4 and Table 5. Through data analysis, it was found that under the excitation conditions of 1 A and 2 A, the acceleration and force of the transducer are the highest at the frequency of 6.4 kHz. The results show that the frequency dependence of the displacement, acceleration and force is same. In conclusion, the transducer must operate under the optimal conditions of prestress, bias magnetic field, and frequency to achieve good output characteristics.

## 5. Conclusions

This study presents a dynamic characteristic model for a giant magnetostrictive transducer working at high frequency. The model can fully describe the effects of the magnetic field, stress, frequency on the output characteristic of the transducer. The output displacement, acceleration, and force are studied by the experimental system. The accuracy and feasibility of the proposed model are tested by the calculated results and the experimental ones. When the driving current is 2 A, and the frequency is 6.4 kHz, the calculated amplitude of the displacement, acceleration, and force are 46 μm, 2000 m/s^2^, and 20.2 N. The experimental values of the average amplitude of displacement, acceleration, and force are 49 μm, 1943 m/s^2^, and 20.2 N. The results show that the modeled responses compare well with the experiments. Moreover, the output displacement, acceleration, and force are analyzed at different frequencies. The results show that the transducer has the best energy conversion rate when it operates at 6.4 kHz frequency.

## Figures and Tables

**Table 1:** Parameter values of the Terfenol-D.

Parameter	Value	Parameter	Value
Young’s modulus	3 × 10^10^ Pa	Relative permeability	9.2
Poisson’s ratio	0.45	Density	9250 kg/m^3^
Heat Expansion Coefficient	12 × 10^−6^ °C^−1^	Curie temperature	380 °C
Relative dielectric constant	1	Conductivity	1.7 × 10^6^ S/m
Resistivity	6 × 10^−7^ Ω·m	Specific heat capacity	0.35 kJ/(kg·K)
Saturation magnetostriction	1555 ppm	Saturation magnetization	6.65 × 10^5^ A/m
Heat conductivity	13.5 W/(m·K)		

**Table 2:** Main materials and parameters of the giant magnetostrictive transducer.

Parameter	Value	Parameter	Value
Terfenol-D rod	2 rods	Coil turns	150 turns
Terfenol-D rod radius	7.5 mm	Coil bobbin length	120 mm
Terfenol-D rod length	102 mm	Coil outward radius	14 mm
Horn top diameter	28 mm	Horn length	310 mm
Horn bottom diameter	67 mm	Shell radius	76 mm
Magnetic yoke length	60 mm	Magnetic yoke width	16 mm

**Table 3:** Experimental data of the output displacement.

Frequency	Current	Voltage	Temperature	Temperature Difference	Amplitude
5.6 kHz	1 A	150 V	21.1 °C	1.1 °C	17 μm
5.6 kHz	2 A	240 V	21.6 °C	1.6 °C	29 μm
5.8 kHz	1 A	143 V	21.3 °C	1.3 °C	18 μm
5.8 kHz	2 A	220 V	21.7 °C	1.7 °C	31 μm
6 kHz	1 A	130 V	21.5 °C	1.5 °C	21 μm
6 kHz	2 A	200 V	21.9 °C	1.9 °C	35 μm
6.2 kHz	1 A	115 V	22.0 °C	2.0 °C	28 μm
6.2 kHz	2 A	190 V	22.2 °C	2.2 °C	41 μm
6.4 kHz	1 A	105 V	22.4 °C	2.4 °C	33 μm
6.4 kHz	2 A	175 V	22.6 °C	2.6 °C	49 μm
6.6 kHz	1 A	95 V	22.6 °C	2.6 °C	26 μm
6.6 kHz	2 A	155 V	22.8 °C	2.8 °C	40 μm
6.8 kHz	1 A	85 V	22.8 °C	2.8 °C	18 μm
6.8 kHz	2 A	140 V	22.9 °C	2.9 °C	32 μm
7 kHz	1 A	77 V	22.9 °C	2.9 °C	15 μm
7 kHz	2 A	125 V	23.0 °C	3.0 °C	26 μm

**Table 4:** Experimental data of the output acceleration.

Frequency	Current	Acceleration	Frequency	Current	Acceleration
5.6 kHz	1 A	570 m/s^2^	5.6 kHz	2 A	1260 m/s^2^
5.8 kHz	1 A	650 m/s^2^	5.8 kHz	2 A	1428 m/s^2^
6 kHz	1 A	778 m/s^2^	6 kHz	2 A	1581 m/s^2^
6.2 kHz	1 A	820 m/s^2^	6.2 kHz	2 A	1700 m/s^2^
6.4 kHz	1 A	925 m/s^2^	6.4 kHz	2 A	1943 m/s^2^
6.6 kHz	1 A	860 m/s^2^	6.6 kHz	2 A	1680 m/s^2^
6.8 kHz	1 A	790 m/s^2^	6.8 kHz	2 A	1400 m/s^2^
7 kHz	1 A	600 m/s^2^	7 kHz	2 A	1100 m/s^2^

**Table 5:** Experimental data of the output force.

Frequency	Current	Force	Frequency	Current	Force
5.6 kHz	1 A	4 N	5.6 kHz	2 A	10 N
5.8 kHz	1 A	5 N	5.8 kHz	2 A	11 N
6 kHz	1 A	6 N	6 kHz	2 A	14 N
6.2 kHz	1 A	8 N	6.2 kHz	2 A	16 N
6.4 kHz	1 A	10 N	6.4 kHz	2 A	20 N
6.6 kHz	1 A	7 N	6.6 kHz	2 A	11 N
6.8 kHz	1 A	6 N	6.8 kHz	2 A	10 N
7 kHz	1 A	5 N	7 kHz	2 A	8 N

## Data Availability

Not applicable.
